# Zebrafish and Medaka: new model organisms for modern biomedical research

**DOI:** 10.1186/s12929-016-0236-5

**Published:** 2016-01-28

**Authors:** Cheng-Yung Lin, Cheng-Yi Chiang, Huai-Jen Tsai

**Affiliations:** Graduate Institute of Biomedical Sciences, Mackay Medical College, No.46, Section 3, Zhongzheng Rd., Sanzhi Dist., New Taipei City, 252 Taiwan

**Keywords:** Disease model organism, Developmental biology, Gene regulation, *in vivo* expression, Transgenic fish

## Abstract

Although they are primitive vertebrates, zebrafish (*Danio rerio*) and medaka (*Oryzias latipes*) have surpassed other animals as the most used model organisms based on their many advantages. Studies on gene expression patterns, regulatory *cis*-elements identification, and gene functions can be facilitated by using zebrafish embryos via a number of techniques, including transgenesis, *in vivo* transient assay, overexpression by injection of mRNAs, knockdown by injection of morpholino oligonucleotides, knockout and gene editing by CRISPR/Cas9 system and mutagenesis. In addition, transgenic lines of model fish harboring a tissue-specific reporter have become a powerful tool for the study of biological sciences, since it is possible to visualize the dynamic expression of a specific gene in the transparent embryos. In particular, some transgenic fish lines and mutants display defective phenotypes similar to those of human diseases. Therefore, a wide variety of fish model not only sheds light on the molecular mechanisms underlying disease pathogenesis *in vivo* but also provides a living platform for high-throughput screening of drug candidates. Interestingly, transgenic model fish lines can also be applied as biosensors to detect environmental pollutants, and even as pet fish to display beautiful fluorescent colors. Therefore, transgenic model fish possess a broad spectrum of applications in modern biomedical research, as exampled in the following review.

## Background

Although zebrafish (*Danio rerio*) and medaka (*Oryzias latipes*) are primitive vertebrates, they have several advantages over other model animals. For example, they are fecund and light can control their ovulation. Spawning takes place frequently and no limitation in their spawning season. Microinjection of fertilized eggs is easily accessible and relatively cheap. Their embryos are transparent, making it easy to monitor the dynamic gene expression in various tissues and organs *in vivo* without the need to sacrifice the experimental subjects. Their genome sizes are approximately 20 to 40 % of the mammalian genome, making them the only vertebrates available for large-scale mutagenesis. Their maturation time takes only 2 ~ 3 months, which is relatively less laborious and time-saving for generating transgenic lines. In addition, many routine techniques of molecular biology and genetics, including knock-in, knockdown and knockout, are well developed in the model fish. Therefore, zebrafish and medaka are new excellent animal systems for the study of vertebrate-specific biology *in vivo*.

## Review

### Germline transmission

The F0 transgenic line can be established once the exogenous gene can be successfully transferred to the embryos, followed by stable germline transmission of the transgene to the F1 generation. Generally, around 10–20 % of treated embryos have a chance to achieve germline transmission [1]. It has been reported that a foreign gene flanked with inverted terminal repeats of adeno-associated virus can be used to enhance the ubiquitous expression and stable transmission of transgene in model fish [2]. Meanwhile, transgenesis can be facilitated by using the Tol2 transposon derived from medaka [3]. Transposase catalyzes transposition of a transgene flanked with the Tol2 sequence [4]. The efficiency of Tol-2-mediated germline transmission could range from 50 to 70 % of injected embryos [4, 5].

A cutting-edge technique has taken the study of fish gene transfer to new horizons, such as knockout zebrafish by the transcription activator-like effector nuclease (TALEN) system and the clustered regularly interspaced short palindromic repeats (CRISPR) combined with CRISPR-associated proteins 9 (Cas9) [6, 7]. The TALEN system involves the DNA recognition domain of transcription activator-like effectors (TALEs) and a nuclease domain for generation of nicks on DNA sequences. The CRISPR/Cas9 system directed by a synthetic single guide RNA can induce targeted genetic knockout in zebrafish. The main difference between these two systems is based on their recognition mechanisms. Unlike the TALEs applied in the TALEN system, the CRISPR/Cas9 system recognizes its target DNA fragment by the complementary non-coding RNA. The development of the TALEN and CRISPR/Cas9 systems provides new genomic editing approaches for establishing genetic knockout fish lines [8].

### Transgenic fluorescent fish

The fluorescence protein gene (FPG) has been widely applied as a reporter gene in studies of the transgene expression by direct visualization under fluorescent microscopy *in vivo* [9]. Many transgenic model fish lines harbor an FPG driven by various tissue-specific promoters, including the erythroid-specific GATA promoter [10], muscle-specific α-actin promoter [11], rod-specific *rhodopsin* promoter [12], neuron-specific *isl*-1 promoter [13], pancreas-specific *pdx*-1 and *insulin* promoters [14], myocardium-specific *cmlc*2 promoter [15], liver-specific L-FABP promoter [16], bone-specific *col10a1* promoter [17], macrophage-specific *mfap4* promoter [18], and germ cell-specific *vasa* promoter [19]. Using medaka β-actin promoter, Tsai’s lab generated a transgenic line of medaka displaying green FP ubiquitously around the whole fish from F0 through F2 generations in a Mendelian inheritance manner [20]. This is known as the first transgenic line of glowing pet fish, which was reported by *Science* [21] and *Far Eastern Economic Review* [22] and honored to be selected as among “The Coolest Inventions of 2003” by *TIME* [23]. The DNA sequences of the afore-mentioned promoters ranging from 0.5 to 6.5 kb are sufficient to drive the FPG reporter to mimic the tissue-specific expression of endogenous gene.

However, some genes require a longer regulatory DNA sequence, such as more than 20 kb, to fully recapitulate the characteristic expression profiles of endogenous genes. In that case, bacterial artificial chromosome (BAC) and phage P1-dereived artificial chromosome (PAC) have been commonly used for this purpose [24]. For example, the zebrafish *rag1* gene, flanked with PAC DNA containing 80 kb at the 5′ upstream and 40 kb at the 3′ downstream, can be expressed specifically in lymphoid cells. Instead of using the tedious Chi-site dependent approach, Jessen et al. reported a two-step method to construct a BAC clone [25]. Employing this protocol, Chen et al. constructed a BAC clone containing the upstream 150 kb range of zebrafish *myf5* and generated a transgenic line *Tg* (*myf5*:*gfp*) [26]. This transgenic line is able to recapitulate the somite-specific and stage-dependent expression of the endogenous *myf*5 at an early developmental stage. In summary, all the above transgenic lines should be very useful materials for studying both gene regulation and cell development.

## New materials for developmental, biological and biomedical studies

### A) Developmental biology

#### Heart development

Zebrafish is particularly useful for studying heart development for the following reasons: (a) Zebrafish have a primitive form of the heart, which is completely developed within 48 h post-fertilization (hpf). (b) The cardiac development can be easily observed in the transgenic line possessing a FP-tagged heart. (c) The zebrafish embryos with a defective cardiovascular system can still keep on growing by acquiring oxygen diffused from water. (d) Discovery of genes involved in heart development can be facilitated by a simple haploid mutation method [27]. For example, using the zebrafish *jekyll* mutant, which has defective heart valves, Walsh and Stainier discovered that UDP-glucose dehydrogenase is required for zebrafish embryos to develop normal cardiac valves [28].

Tsai’s lab is the first group to generate a transgenic zebrafish line that possesses a GFP-tagged heart [15]. This line was established from zebrafish embryos introduced with an expression construct, in which the GFP reporter is driven by an upstream control region of zebrafish *cardiac myosin light chain 2* gene (*cmlc*2). Using this transgenic line, Raya et al. found that the Notch signaling pathway is activated is during the regenerative response [29]. Shu et al. reported that Na, K-ATPase α1B1 and α2 isoforms have distinct roles in the patterning of zebrafish heart [30]. This transgenic line should also be useful for studying the dynamic movement and cell fate of cardiac primordial cells. For example, Forouhar et al. proposed a hydro-impedance pump model for the embryonic heart tubes of zebrafish [31]. A 4D dynamic image of cardiac development has been developed [32]. Furthermore, Hami et al. reported that a second heart field is required during cardiac development [33]. Thus, recently, Nevis et al. stated that *tbx1* plays a function for proliferation of the second heart field, and the zebrafish *tbx1*-null mutant resemble the heart defects in DiGeorge syndrome [34].

Thus, the expression pattern of heart-specific genes could be analyzed based on heart progenitor cells collected in this transgenic line. The analysis of gene or protein expression dynamics at different developmental stages could also be conducted. Furthermore, this transgenic fish is a potential platform for detecting chemicals, drugs and environmental pollutants affecting heart development, as detailed in following section.

#### Muscle development

*In vivo* transient assay of the injected DNA fragments in model fish embryos is a simple yet effective way to analyze the function of regulatory *cis*-elements. For example, Myf5, one of myogenesis regulatory factors (MRF), plays key roles in the specification and differentiation of muscle primordial cells during myogenesis. The expression of *myf5* is somite-specific and stage-dependent, and its activation and repression are delicately orchestrated. Using *in vivo* transient assay, Chen et al. found that a novel *cis*-element located at −82/-62 is essential for somite-specific expression of *myf*5 [35]. Lee et al. revealed that this −82/-62 *cis*-element is specifically bound by Forkhead box D3, and proposed that somite development is regulated by the Pax3-FoxD3-Myf5 axis [36]. Besides FoxD3, FoxD5, another protein in the Forkhead box family, is necessary for maintaining the anterior-posterior polarity of somite cells in mesenchymal-epithelial transition [37]. The expression of FoxD5 is regulated by Fgf signaling in anterior pre-somitic mesoderm (PSM), which indicates that Fgf-FoxD5-Mesp signaling takes place in somitogenesis [37]. Furthermore, analysis of the loci of adjacent *mrf4* and *myf5* revealed the complicated regulation mechanism of the MRF genes. It was also found that the biological function of Mrf4 is related to myofibril alignment, motor axon growth, and organization of axonal membrane [38].

**Fig. 1:**
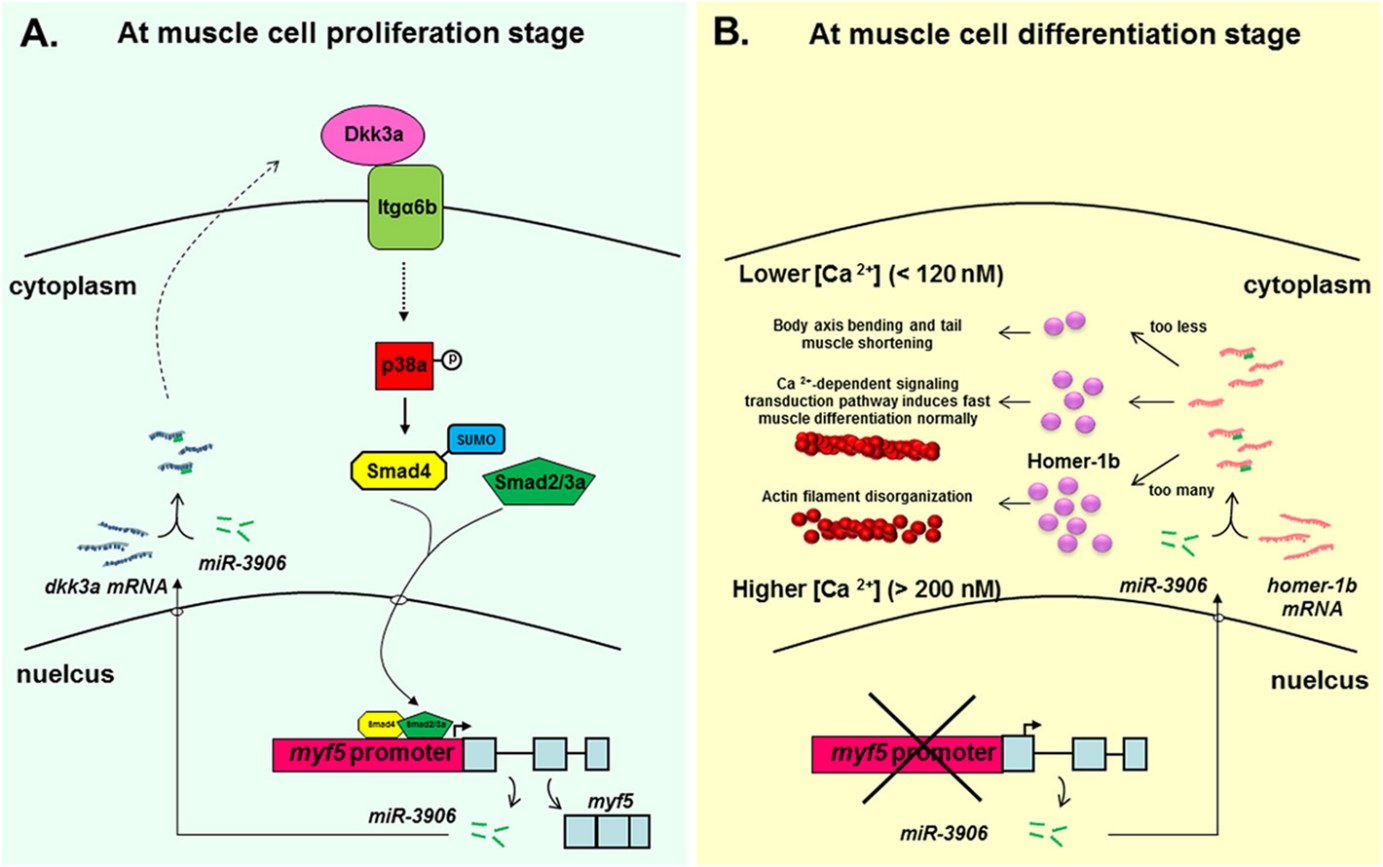
*miR*-*3906* **silences different target genes at different muscle developmental stages. a** At the early muscle development, Dkk3a interacts with its receptor Itgα6b, resulting in the phosphorylation of p38a and the formation of the Smad2/3a/4 complex, which in turn, activates the *myf5* promoter activity. When *myf5* is highly transcribed, the intronic *miR*-*3906* suppresses the transcription of *myf5* through silencing the Dkk3a [39, 41–43]. **b** At the late muscle development, *miR*-*3906* starts transcription at its own promoter and switches to silence *homer1b* to control the homeostasis of intracellular calcium concentration ([Ca^2+^]_i_) in fast muscle cells [40]. Either *miR*-*3906*-knockdown or *homer*-*1b*-overexpression causes the increase of Homer-1b protein, resulting in an enhanced level of [Ca^2+^]_i_, which in turn, disrupts sarcomeric actin filament organization. In contrast, either *miR*-*3906*-overexpression or *homer*-*1b*-knockdown causes the decrease of Homer-1b, resulting in a reduced [Ca^2+^]_i_ and thus a defective muscle phenotype [40]

The molecular mechanism that underlies the repression of *myf5* has also been reported. For example (Fig. 1a), a strong repressive element of zebrafish *myf5* was found within intron I (+502/+835) [39]. This repressive element is modulated by a novel intronic microRNA, termed *miR*-*In300* or *miR*-*3906* [40]. When *myf5* transcripts reach the highest level after specification, the accumulated *miR*-*3906* starts to reduce the transcription of *myf5* through silencing the positive factor Dickkopf-related protein 3 (Dkk3r or Dkk3a) for the *myf5* promoter [41]. Itgα6b is a receptor of secretory Dkk3a and that interaction between Itgα6b and Dkk3a is required to drive the downstream signal transduction which regulates *myf5* promoter activity in somite during embryogenesis of zebrafish [42]. Dkk3a regulates p38a phosphorylation to maintain Smad4 stability, which in turn enables the formation of the Smad2/3a/4 complex required for the activation of the *myf5* promoter [43]. However, when *myf5* transcripts are reduced at the later differentiation stage, *miR*-*3906* is able to be transcribed by its own promoter [40] (Fig. 1b). Furthermore, increased expression of *miR*-*3906* controls the intracellular concentration of Ca^2+^ ([Ca^2+^]_i_) in fast muscle cells through subtly reducing *homer*-*1b* expression. The homeostasis of [Ca^2+^]_i_ is required during differentiation to help maintain normal muscle development [40]. Nevertheless, it remains to be investigated how *miR*-*3906* switches its target gene at different developmental stages.

Apart from the regulation of somitogenesis, Myf5 is also involved in craniofacial muscle development. The functions of Myf5 in cranial muscles and cartilage development are independent of Myod, suggesting that Myf5 and Myod are not redundant. Thus, three possible pathways could be associated with the molecular regulation between Myf5 and Myod: (i) Myf5 alone is capable of initiating myogenesis, (ii) Myod initiates muscle primordia, which is subdivided from the *myf5*-positive core, and (iii) Myod alone, but not Myf5, modulates the development of muscle primordia [44]. Furthermore, the *six1a* gene was found to play an important role in the interaction between Myf5 and Myod [45]. In cartilage development, *myf5* is expressed in the paraxial mesoderm at the gastrulation stage. Myf5 plays a role in mesoderm fate determination by maintaining the expression of *fgf3*/*8*, which in turn, promotes differentiation from neural crest cells to craniofacial cartilage [46]. This research on *myf5* not only reveals that it has different functions between craniofacial muscle development and somitogenesis, but it also opens up a new study field for understanding craniofacial muscle development.

Hinits et al. reported no phenotype both in *myf5*-knockdown embryos and *myf5*-null mutant, suggesting that Myf5 is rather redundant in somitogenesis of zebrafish [47]. However, it is hard to reasonably explain why these embryos and mutant are all lethal and can’t grow to adulthood. On the other hand, Lin et al. reported an observable defective phenotype in *myf5*-knockdown embryos, and claimed that the concentration of *myf5*-MO they used can inhibit maternal *myf5* mRNA translation [46]. This discrepancy might attribute the effectiveness of MO used or the different phenotypes between the knockdown embryos and knockout mutant in this case.

### Ocular and nerve development

The retina-specific expression of the carp *rhodopsin* gene is controlled by two upstream regulatory DNA *cis*-elements [48]. One is located at −63 to −75, which is the carp neural retina leucine zipper response-like element; the other is located at −46 to −52, which is a carp-specific element crucial to reporter gene expression in medaka retinae. Intriguingly, immediate activation of early growth response transcriptional regulator Egr1 could result in the incomplete differentiation of retina and lens, leading to microphthalmos [49]. Another important factor for ocular development is the ADP-ribosylation factor-like 6 interacting protein 1 (Arl6ip1). Loss of Arl6ip1 function leads to the absence of retinal neurons, disorganized retinal layers and smaller optic cups [50]. Upon losing Arl6ip1, retinal progenitors continued to express cyclin D1, but not *shh* or *p57kip2*, suggesting that eye progenitor cells remained at the early progenitor stage, and could not exit the cell cycle to undergo differentiation [51]. Additionally, it has been reported that Arl6ip1 is essential for specification of neural crest derivatives, but not neural crest induction. Tu et al. found that Arl6ip1 mutation causes abnormal neural crest derivative tissues as well as reduced expression of neural crest specifier genes, such as *foxd3*, *snail1b* and *sox10*, indicating that Arl6ip1 is involved in specification, but not induction, of neural crest cells [52]. Furthermore, they found that Arl6ip1 could play an important role in the migration of neural crest cells because in the *arl6ip1*-knockdown embryos, *crestin*- and *sox10*-expressing neural crest cells failed to migrate ventrally from neural tube into trunk. More recently, Lin et al. found that Ras-related nuclear (Ran) protein is conjugated with Arl6ip1, and proposed that Ran protein associates with Arl6ip1 to regulate the development of retinae [53].

To date, no *in vivo* model system has been established to identify cells in the CNS that can specifically respond with regeneration after stresses, and, even if identified, no method is in place to trace these responsive cells and further identify their cell fates during hypoxic regeneration. To address these issues, Lee et al. generated a transgenic zebrafish line *huORFZ*, which harbors the upstream open reading frame (uORF) from human CCAAT/enhancer-binding protein homologous protein gene (*chop*), fused with the GFP reporter and driven by a cytomegalovirus promoter [54]. After *huORFZ* embryos were treated with heat-shock or under hypoxia, the GFP signal was exclusively expressed in the CNS, resulting from impeding the huORF^*chop*^-mediated translation inhibition [54]. Interestingly, Zeng et al. found that GFP-(+) cells in spinal cord respond to stress, survive after stress and differentiate into neurons during regeneration (Chih-Wei Zeng, Yasuhiro Kamei and Huai-Jen Tsai, unpublished data).

### MicroRNAs and development

MicroRNAs (miRNAs) are endogenous single-stranded RNA molecules of 19–30 nucleotides (nt) that repress or activate the translation of their target genes through canonical seed- and non-canonical centered miRNA binding sites. The known mechanisms involved in miRNAs-mediated gene silencing are decay of mRNAs and blockage of translation [55–57]. Probably the expression of 30 ~ 50 % of human genes is regulated by miRNAs [58, 59]. Therefore, to understand gene and function in cells or embryos, it is important to exactly know the target gene(s) of a specific miRNA at different phase of cells or at particular stages of developing embryos.

Instead of using a bioinformatic approach, the Tsai’s lab developed the Labeled miRNA Pull-down (LAMP) assay system, which is a simple but effective method to search for the candidate target gene(s) of a specific miRNA under investigation [60]. LAMP assay system yields fewer false-positive results than a bioinformatic approach. Taking advantage of LAMP, scientists discovered that *miR*-*3906* silences different target genes at different developmental stages, e.g., at early stage, *miR*-*3906* targets *dkk3a* [41], while at late stage, it targets *homer*-*1b* [40] (Fig. 1).

**Fig. 2:**
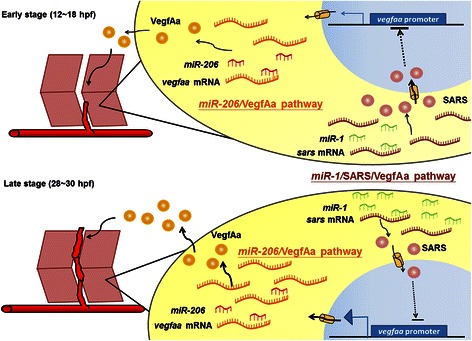
*miR-1* and *miR-206* silence different target genes and play opposing roles in zebrafish angiogenesis. Both *miR*-*1* and *miR*-*206* are muscle-specific microRNAs and share identical seed sequences. However, they silence different target genes to affect the secreted VegfAa level through different pathways [62]. The *miR*-*1*/SARS/VegfAa pathway plays a positive role in angiogenesis since SARS, a negative factor for VegfAa promoter transcription, is silenced by *miR*-*1*, resulting in the increase of VegfAa. However, the *miR*-*206*/VegfAa pathway plays a negative role since VegfAa is silenced directly by *miR*-*206*. Dynamic changes of *miR*-*1* and *miR*-*206* levels are also observed [62]. The *miR*-*1* level gradually increases between 12 and 20 hpf and significantly increases further between 20 and 32 hpf, while the *miR*-*206* level is only slightly changed during this same period. Consequently, VegfAa increases greatly from 24 to 30 hpf, which might be responsible for the continuous increase of *miR*-*1*/SARS/VegfAa pathway, but not *miR*-*206*/VegfAa pathway. Therefore, temporal regulation of the expression of *miR*-*1* and *miR*-*206* with different target genes occur during embryonic angiogenesis in somitic cells of zebrafish

In another example (Fig. 2), *miR*-*1* and *miR*-*206* are two muscle-specific microRNAs sharing the same seed sequences. They are able to modulate the expression of vascular endothelial growth factor Aa (VegfAa) and serve as cross-tissue signaling regulators between muscle and vessels. Since *miR*-*1* and *miR*-*206* share identical seed sequences, Stahlhut et al. demonstrated that they can silence the same target gene, such as *vegfaa*, and considered them as a single cross-tissue regulator termed as *miR*-*1*/*206* [61]. *miR*-*1*/*206* reduces the level of VegfAa, resulting in the inhibition of the angiogenic signaling [61]. Surprisingly, using the LAMP assay system, Lin et al. reported that the target genes for *miR*-*1* and *miR*-*206* are different [62]. While *miR*-*206* targets *vegfaa*, *miR*-*1* targets seryl-tRNA synthetase gene (*sars*). SARS is a negative regulator of VegfAa. Although both *miR*-*1* and *miR*-*206* have identical seed sequences, the *sars*-3′UTRs of zebrafish, human and mouse origins can be recognized only by *miR*-*1* in zebrafish embryos and mammalian cell lines (HEK-293 T and C2C12), but not by *miR*-*206* [62]. Conversely, the *vegfaa*-3′UTR is targeted by *miR*-*206*, but not by *miR*-*1*. Therefore, Lin et al. concluded that *miR*-*1* and *miR*-*206* are actually two distinct regulators and play opposing roles in zebrafish angiogenesis. The *miR*-*1*/SARS/VegfAa pathway promotes embryonic angiogenesis by indirectly controlling VegfAa, while *miR*-*206*/VegfAa pathway plays an anti-angiogenic role by directly reducing VegfAa. Interestingly, they also found that the *miR*-*1*/SARS/VegfAa pathway increasingly affects embryonic angiogenesis at late developmental stages in somitic cells [62]. It remains to be studied how *miR*-*1* increases in abundance at late stage.

### miRNAs in regeneration

Different from mammals, zebrafish have the ability to regenerate injured parts in the CNS. Many miRNAs have been found in the CNS. Since miRNAs are involved in many aspects of development and homeostatic pathways, they usually play important roles in regeneration [63]. It has been shown that several miRNAs have prominent functions in regulating the regeneration process. For example, *miR*-*210* promotes spinal cord repair by enhancing angiogenesis [64], and the *miR*-*15* family represses proliferation in the adult mouse heart [65]. Furthermore, miRNAs *miR*-*29b* and *miR*-*223* are identified following optic nerve crush. By gene ontology analysis, *miR*-*29b* and *miR*-*223* are found to regulate genes, including *eva1a*, *layna*, *nefmb*, *ina*, *si*:*ch211*-*51a6.2*, *smoc1*, and *sb*:*cb252*. These genes are involved in cell survival or apoptosis, indicating that these two miRNAs are potential regulators of optic nerve regeneration [66].

### Hematopoiesis

Although the main hematopoietic sites in zebrafish differ from those in mammals, both zebrafish and mammals share all major blood cell types that arise from common hematopoietic lineages [67]. Moreover, many genes and signaling pathways involved in hematopoiesis are conserved among mammals and zebrafish. For example, *scl*, one of the first transcription factors expressed in early hematopoietic cells, is evolutionarily conserved. During definitive hematopoiesis, *runx1* marks hematopoietic stem cells (HSCs) in both mouse and fish. Additionally, in differentiated populations, *gata1*, the erythroid lineage regulator, *pu.1* and *c*/*ebp*, the myeloid lineage regulators, and *ikaros*, a mark of the lymphoid population, are in accordance with the hematopoietic hierarchy in zebrafish and mammals [68]. Thus, the findings with respect to zebrafish blood development could be applied to mammalian system.

Genetic screening in zebrafish has generated many blood-related mutants that help researchers understand hematopoietic genes and their functions [69]. For example, the *spadetail* mutant carrying a mutated *tbx16* exhibits defective mesoderm-derived tissues, including blood. This mutant displays the decrease levels *tal1*, *lmo2*, *gata2*, *fli1* and *gata1* in the posterior lateral mesoderm, indicating the important role of *tbx16* during hemangioblast regulation [70]. Chemical screening in zebrafish using biologically active compounds is also a powerful approach to identify factors that regulate HSCs. For example, it is well known that prostaglandin (PG) E2 increases the induction of stem cells in the aorta–gonad–mesonephros region of zebrafish, as demonstrated by increasing expressions of *runx1* and *cmyb*, which, in turn, increases engraftment of murine marrow in experimental transplantation [71]. In human clinical trials, the treatment of cord blood cells with dimethyl PGE2 caused an increase in long-term engraftment [72], suggesting that a compound identified in zebrafish could have clinical application in humans.

### B) Zebrafish as an animal model for human diseases

#### Cardiovascular diseases

Model fish are excellent materials for the study of human diseases due to some mutants display similar phenotypes of human diseases [73]. In addition, essential genes and thereof regulation to control the development of tissues or organs are highly conserved [74]. For example, Tbx5 is a T-box transcription factor responsible for cell-type specification and morphogenesis. The phenotypes of *tbx5* mutant are highly similar among mammals and zebrafish. Thus, transgenic fish with heart-specific fluorescence could provide a high-through screening platform for drugs for cardiovascular disease. For example, the Tsai’s lab established a transgenic line which could be induced to knock down the expression level of cardiac troponin C at any developmental stage, including embryos, larva or adult fish. The reduction of troponin C resulted in mimicry of dilated cardiomyopathy, and the incomplete atrioventricular blocking disease in humans. Therefore, this transgenic line is expected to make a significant contribution to drug screening and the elucidation of the molecular mechanisms underlying cardiovascular diseases. Next, the effect of drugs on embryonic development was also studied.

Amiodarone, which is a class III antiarrhythmic agent, is being used for the treatment of tachyarrhythmia in humans. However, Amiodarone-treated zebrafish embryos were found to exhibit backflow of blood in the heart [75]. Subsequent research showed that Amiodarone caused failure of cardiac valve formation [75]. Specifically, Amiodarone induces ectopic expression of *similar to versican b* (*s*-*vcanb*), resulting in repression of EGFR/GSK3β/Snail signaling, which in turn, upregulates *cdh5* at the heart field, and causes defective cardiac valves [76]. Moreover, Amiodarone was found to repress metastasis of breast cancer cells by inhibiting the EGFR/ERK/Snail pathway [77], a phenomenon analogous to the inhibitory effects of Amiodarone on EMT transition observed in the heart.

Last but not least, although zebrafish has a two-chambered heart, relative to mouse, rat, and rabbit, its heart rate, action potential duration (APD) and electrocardiogram (ECG) morphology are similar to those of humans. [78, 79]. Additionally, Tsai et al. demonstrated that the *in vitro* ECG recording of zebrafish heart is a simple, efficient and high throughput assay [80]. Thus, zebrafish can serve as a platform for direct testing of drug effect on APD prolongation and prolonged QT interval, which is required by the FDA as a precondition for drug approval.

#### Cancer study: melanoma

Zebrafish become a popular experimental animal for the studies of human cancer [81], in part because the fish homologs of human oncogenes and tumor suppressor genes have been identified, and in part because signaling pathways regulating cancer development are conserved [82–84]. Amatruda et al. reported that many zebrafish tumors are similar to those of human cancer in the histological examination [85]. The zebrafish transgenic line with skin-specific red fluorescence could be applied for skin tumor detection [86]. When the embryos of this line were treated with solutions containing arsenic, the tumors induced on the skin could be easily identified by naked eye under fluorescent microscope. Therefore, this transgenic line can be potentially used for the study of skin diseases. For example, the common skin cancer melanoma may be screened by the red fluorescence expression in this transgenic line.

Zebrafish transgenic line could also be applied to establish models simulating melanoma development. The human oncogenic *BRAF*^*V600E*^ was expressed under the control of the zebrafish melanocyte *mitfa* promoter to establish a melanoma model [87]. Combining skin-specific red fluorescence with *mitfa*-driven oncogene expression, the melanoma could be easily traced. Therefore, transgenic lines and mutants of model fish could provide abundant resources for mechanistic studies and therapeutic research in human diseases.

#### Cancer study: metastasis

Metastasis involves processes of sequential, interlinked and selective steps, including invasion, intravasation, arrest in distant capillaries, extravasation, and colonization [88]. Zebrafish is again an alternative organism for *in vivo* cancer biology studies. In particular, xenotransplantation of human cancer cells into zebrafish embryos serves as an alternative approach for evaluating cancer progression and drug screening [89]. For example, human primary tumor cells labeled with fluorescence have already been implanted in zebrafish liver, and the invasiveness and metastasis of these cells were directly observable and easily traceable [90]. To investigate the mechanism of local cancer cell invasion, human glioblastoma cells labeled with fluorescence were infiltrated into the brain of zebrafish embryos. It was observed that the injected cells aligned along the abluminal surface of brain blood vessels [91]. By grafting a small amount of highly metastatic human breast carcinoma cells onto the pericardial membrane of zebrafish embryos at 48 hpf, tumor cells were observed to move longitudinally along the aorta [92]. Similarly, highly metastatic human cancer cells labeled with fluorescence were injected into the pericardium of 48-hpf embryos. Afterwards, it is possible to visualize how cancer cells entered the blood circulation and arrested in small vessels in head and tail [93]. In another example, zebrafish embryos were injected with tumorigenic human glioma stem cells at different stages of metastasis, including beginning, approaching, clustering, invading, migrating, and transmigrating [94]. Thus, grafting a small number of labeled tumor cells into transparent zebrafish embryos allows us to dynamically monitor the cancer cells without the interference of immune suppression.

Apart from its utility in analyzing the mechanisms of tumor dissemination and metastasis, the zebrafish model can also be applied to screen potential anticancer compounds or drugs. In addition, zebrafish feature such advantages as easy gene manipulation, short generation cycle, high reproducibility, low maintenance cost, and efficient plating of embryos [95, 96]. Therefore, this small fish is second only to SCID and nude mice as xenograft recipients of cancer cells.

#### Cancer study: leukemia

Leukemia is a cancer related to hematopoiesis. Most often, leukemia results from the abnormal increase of white blood cells. However, some human cancers of bone marrow and blood origins have their parental cells from other blood cell types. The search for efficacious therapies for leukemia is ongoing. Interestingly, the developmental processes and genes related to hematopoiesis are similar between zebrafish and humans, making zebrafish a feasible model for the study of leukemia. In addition, gene expression in zebrafish could be conveniently modified by several approaches, e.g., MO-induced gene knockdown, TALENs and CRISPR/Cas9 gene knockout, and DNA/RNA introduced overexpression [6, 7]. In the study of Yeh et al. [97], the zebrafish model was applied to screen for chemical modifiers of AML1-ETO, an oncogenic fusion protein prevalent in acute myeloid leukemia (AML). Treatment of zebrafish with chemical modifiers of AML1-ETO resulted in hematopoietic dysregulation and elicited a malignant phenotype similar to human AML. Cyclooxygenase-2 (COX2) is an enzyme causing inflammation and pain. Nimesulide is an inhibitor of COX2 and an antagonist to AML1-ETO in hematopoietic differentiation.

FMS-like tyrosine kinase 3 (FLT3) is a class III receptor tyrosine kinase which is normally expressed in human hematopoietic stem and progenitor cells (HSPCs) [98]. Internal tandem duplication (ITD), which may occur at either the juxtamembrane domain (JMD) or the tyrosine kinase domain (TKDs) of FLT3, is observed in one-third of human AML. Zebrafish Flt3 shares an overall 32, 35, and 34% sequence identity with that of human, mouse, and rat, respectively. However, the JMD and the activation loops of TKD are highly conserved, implicating that the functions of FLT3 signaling are evolutionally conserved. Overexpression of human FLT3-ITD in zebrafish embryos induces the ectopic expansion of FLT3-ITD positive myeloid cells. If those embryos are treated with AC220, a potent and relatively selective inhibitor of FLT3, FLT3-ITD myeloid expansion is effectively ameliorated [99]. In another example, isocitrate dehydrogenase (IDH) 1 and 2 are involved in citric acid cycle in intermediary metabolism. IDH mutations are found in approximately 30 % of cytogenetically abnormal AML, suggesting a pathogenetic link in leukemia initiation [100, 101]. Injection of either human IDH1-R132H or zebrafish *idh1*-R146H, a mutant corresponding to human IDH1-R132H, resulted in increased 2-hydroxyglutarate, which in turn induced the expansion of primitive myelopoiesis [102]. Taken together, these reports suggest that the molecular pathways involved in leukemia are conserved between humans and zebrafish. Based on the aforementioned experimental evidence, zebrafish can be an exceptional platform for mimicking human myelodysplastic syndromes and establishing an *in vivo* vertebrate model for drug screening.

#### Cancer study: hepatoma

Several liver tumor models have been reported by liver-specific expression of transgenic oncogenes such as *kras*, *xmrk* and *myc*. These transgenic lines of zebrafish usually generate liver tumors with various severity from hepatocellular adenoma (HCA) to hepatocellular carcinoma (HCC) [103–105]. These three transgenic liver cancer models have been used to identify differentially expressed genes through RNA-SAGE sequencing. For example, researchers have searched genes either up- or downregulated among the three tumor models and analyzed the possible signaling pathways. Then, correlation between zebrafish liver tumor signatures and the different stages of human hepatocarcinogenesis was determined [106]. High tumor incidence and convenient chemical treatment make this inducible transgenic zebrafish a plausible platform for studying on liver tumor progression, regression, and anticancer drug screening.

### Behavioral studies

Interestingly, zebrafish become a modern organism for studying on depressive disorders [107–109]. Because the physiological (neuroanatomical, neuroendocrine, neurochemical) and genetic characteristics of zebrafish are similar to mammals, zebrafish are ideal for high-throughput genetic and chemical genetic screening. Furthermore, since behavioral test of zebrafish for cognitive, approach-avoidance, and social paradigms are available, the identification of depression-like indices in response to physiological, genetic, environmental, and/or psychopharmacological alterations is feasible [110]. Actually, zebrafish display highly robust phenotypes of neurobehavioral disorders such as anxiety-like and approach–avoidance behaviors. Furthermore, novel information of behavioral indices can be exposed, including geotaxis via top-bottom vertical movement [111]. Zebrafish behavior can also be monitored using automated behavioral tracking software, which enhances efficiency and reduces interrater variance [112]. Additionally, zebrafish offer a potential insight into the social aspects of depression [113] and may be suitable for studying the cognitive deficits of depression [114] and its putative etiological pathways [115]. Last but not least, zebrafish are highly sensitive to psychotropic drugs, such as antidepressants, anxiolytics, mood stabilizers, and antipsychotics [116–118], serving as an important tool for drug discovery.

### C) Biosensor for environmental toxicants

Aromatic hydrocarbons, heavy metals and environmental estrogens are currently being used to test the impact of environmental pollutants on animals [119]. These studies mainly focused on mortality and abnormality rates. However, the developing embryos may have already been damaged in a subtle way that would have precluded direct observation of morphology and detection of mortality. To overcome this drawback, transgenic fish can be used because they are designed to study (a) whether toxicants cause defective genes during embryogenesis; (b) whether pollutants affect the expression of tissue-specific gene; and (c) whether the impact of pollutants on embryonic development is dosage dependent. Pollutants can be directly detected by simply observing the coloration change of cells before or after the pollutants can cause morphological damage. Therefore, transgenic model fish are promising organisms for use as bioindicators to environmental toxicants and mutagens [120, 121]. In addition, Chen and Lu reported that the environmental xenobiotics can be detected by a transgenic line of medaka carrying a GFP reporter driven by cytochrome *P450 1a* promoter (*CYP1A*-GFP) [122]. Furthermore, the environmental xenoestrogenic compounds can be specifically detected by a hybrid transgenic line derived from crossing between line *CYP1A*-GFP and line VG-Lux whose Lux reporter activity is driven by a vitellogenin promoter [123]. Lee et al. reported another zebrafish transgenic line, termed *huORFZ* [54], as it has been described in pervious section . At normal condition, the translation of the transferred huORF^*chop*^-gfp mRNA in *huORFZ* embryos is completely suppressed by an inhibitory uORF of human *chop* mRNA (huORF^*chop*^). However, when the *huORFZ* embryos were under ER stress, such as heat shock, cold shock, hypoxia, metals, alcohol, toxicants or drugs, the downstream GFP became apparent due to the blockage of huORF^*chop*^-mediated translation inhibition. Therefore, *huORFZ* embryos can be used to study the mechanism of translational inhibition. Additionally, *huORFZ* embryos can serve a living material to monitor the contamination of hazardous pollutants [124]. Besides the universal *huORFZ* system, zebrafish could also be indicators for specific pollutants. For example, Xu et al. reported a transgenic zebrafish *Tg* (*cyp1a*:*gfp*) which can serve as an *in vivo* assay for screening xenobiotic compounds, since Cyp1a is involved in the aryl hydrocarbon receptor pathway, and can be induced in the presence of Dioxins/Dioxin-like compounds and polycyclic aromatic hydrocarbons [125]. Additional advantages of zebrafish include the small size, abundant number, rapid development and transparent eggs. These features make this model fish more accessible for the studies of molecular toxicology.

## Conclusion

It is increasingly clear that the transgenic fish model is a powerful biomaterial for the studies of multiple disciplines, including molecular biology, developmental biology, neurobiology, cancer biology and regenerative medicine. It provides a simple, yet effective, *in vivo* approach to identify regulatory DNA sequences, as well as determine gene function and molecular pathways. More importantly, an increasing number of papers have reported that (a) the defective phenotype of mutants of model fish can photocopy with known human disorders; and (b) drugs have similar effects on zebrafish and mammalian systems. Therefore, the transgenic fish model offers a useful platform for high-throughput drug screening in biomedical sciences. Additionally, it can serve as an environmental indicator for detecting pollutants in our daily lives.

Nevertheless, there are several limitations and caveats of this fish model. First, unlike mammals, fish lack the heart septation, lung, mammary gland, prostate gland and limbs, which make the fish model impossible for studies of these tissues and organs. Additionally, fish are absent of placenta so that fish embryos are directly exposed to the environment (e.g., drugs or pollutants) without involving the placenta. Second, fish are poikilothermic and usually maintained below 30 °C, which may not be optimal for those mammalian agents adapted for 37 °C in evolution. Last, since the zebrafish genome is tetraploid, it is less straight forward to conduct loss-of-function studies for certain genes.
